# Issue analysis: key characteristics approach for identifying endocrine disruptors

**DOI:** 10.1007/s00204-023-03568-3

**Published:** 2023-08-12

**Authors:** Christopher J. Borgert

**Affiliations:** University of Florida College of Veterinary Medicine, Gainesville, USA

## Abstract

For more than a decade, weight of evidence (WoE) evaluations have been the standard method for determining whether a chemical meets the definition of an endocrine disrupting chemical (EDC). WoE methods consider all data pertinent to satisfying the EDC definition and evaluating those data with respect to relevance, reliability, strength, and coherence with established endocrine physiology and pharmacology. A new approach for identifying EDC hazards has been proposed that organizes and evaluates data according to ten so-called “Key Characteristics (KCs) of EDCs”. The approach claims to address the lack of a widely accepted, systematic approach for identifying EDC hazards, but completely ignores the WoE literature for EDCs. In contrast to WoE methods, the KC approach fails to apply the consensus definition of EDC and is not amenable to empirical testing or validation, is fungible and ensures inconsistent and unreliable results, ignores principles of hormone action and characteristics of dose–response in endocrine pharmacology and toxicology, lacks a means of distinguishing endocrine-mediated from non-endocrine mediated mechanisms, lacks a means to reach a negative conclusion about a chemical’s EDC properties or to distinguish EDCs from non-EDCs, and provides no means for developing a valid consensus among experts nor provides a means of resolving conflicting interpretations of data. Instead of shortcuts like the KC approach, which are prone to bias, error, and arbitrary conclusions, identifying EDCs should rely on WoE evaluations that supply the critical components and scientific rigor lacking in the proposed KCs for EDCs.

## Commentary

An endocrine disrupting chemical (EDC) is a chemical that (1) causes an adverse effect, and (2) causes that effect through an endocrine mode of action (MoA). Authors and organizations that may differ on some details regarding endocrine disruption nonetheless concur on its essential elements, which are captured in that definition (World Health Organization [WHO] 2002; OECD 2012, 2018; Mihaich et al. 2017; Borgert 2022; Marty et al. 2018; ECETOC 2009). For more than a decade, weight of evidence (WoE) evaluations have been the standard method for determining whether a chemical meets that definition (World Health Organization [WHO] 2002; U.S. EPA 2011; OECD 2012). WoE methods consider all data pertinent to satisfying the EDC definition and evaluating those data with respect to relevance, reliability, strength, and coherence with established endocrine physiology and pharmacology (Rhomberg et al. 2010; Borgert et al. 2011, 2014; Rhomberg 2014; Lutter et al. 2015; Bridges and Solomon 2016; Mihaich et al. 2017; Neal et al. 2017; Mihaich and Borgert 2018; Borgert 2022).

Because experimental proof of causation may be impossible, WoE methods strive to link adverse effects to endocrine MoAs by objective reasoning applied to relevant data through a series of transparent steps. The steps in a WoE evaluate whether a chemical can operate through an endocrine mechanism and whether that endocrine mechanism is then responsible for an adverse effect(s) of the chemical. Of critical importance is that WoE methods provide a transparent means to resolve conflicts in the data through comparisons to known positive and negative controls for established endocrine MoAs (Rhomberg et al. 2010; Borgert et al. 2011, 2014; Rhomberg 2014; Lutter et al. 2015; Bridges and Solomon 2016; Mihaich et al. 2017; Neal et al. 2017; Mihaich and Borgert 2018; Borgert 2022).

Recently, a new approach for identifying EDC hazards has been proposed that organizes and evaluates data according to ten so-called “Key Characteristics (KCs) of EDCs” (La Merrill et al. 2020). The approach claims to address the lack of a widely accepted, systematic approach for identifying “EDC hazards,” but completely ignores the WoE literature cited here (Rhomberg et al. 2010; Borgert et al. 2011, 2014; Rhomberg 2014; Lutter et al. 2015; Bridges and Solomon 2016; Mihaich et al. 2017; Neal et al. 2017; Mihaich and Borgert 2018; Borgert 2022). Proponents of the KC approach define EDCs as “exogenous chemicals that interfere with hormone action, thereby increasing the risk of adverse health outcomes, including cancer, reproductive impairment, cognitive deficits and obesity” and use chemicals alleged to be EDCs to illustrate the “KC-based approach.” In fact, the KC approach searches for and organizes data according to broad, mechanistically indistinct categories such as “alters hormone metabolism or clearance,” and “alters signal transduction in hormone-responsive cells,” which are phenomena that occur by endocrine as well as non-endocrine mechanisms (Marty et al. 2018). It then accepts those data as evidence of EDC properties without evaluating whether the chemical alters any specific endocrine MoA. Thus, the KC approach does not organize and evaluate data to address the two criteria (endocrine MoA that causes an adverse effect) required by the definition of EDC. More problematic, however, is that when used “…to identify EDC hazards,” the KC approach circumvents the definition altogether.

The KC approach for EDCs is based on the claimed success of ten Key Characteristics for identifying carcinogens, (Smith et al. 2016) a method that has been shown to be no more predictive or informative than the toss of a coin (Becker et al. 2017). Advocates of the KC approach for EDCs assert that it is grounded on established mechanisms of hormone action, yet also argue that, like the KC approach for carcinogens, the KC approach for EDCs “frees the reviewer” from linking specific endocrine MoAs to adverse effects (La Merrill et al. 2020). That assertion not only defies logic, but it also contradicts the definition of an EDC. If the KC approach were firmly grounded in mechanistic understanding, it would facilitate and enable establishing the link between an endocrine MoA and adverse effects without any need to free assessors from that requirement.

Although the KC approach lacks the scientific rigor of WoE standards, it is touted to be faster, easier, and less restrictive than WoE methods. Some advocates of the KC approach for EDCs defend its lack of rigor on the premise that it is merely a systematic means of organizing the data regarding the EDC properties of a chemical and they contend that it is not a check-box approach for identifying EDCs (La Merrill et al. 2020). Yet, despite lacking required elements that seem obvious for systematic approaches–i) criteria for searching and selecting data that address the requirements of the EDC definition; ii) criteria for evaluating data quality, relevance and reliability; iii) a transparent means to resolve contradictions among the data–the KCs are, in fact, applied in a check-box fashion and used to reach conclusions regarding a chemical’s EDC status (Muñoz et al. 2020).

The author, in collaboration with members of the Endocrine Policy Forum (EPF), evaluated articles published in peer-reviewed journals through the end of 2020 reporting on the Key Characteristics approach for EDCs (La Merrill et al. 2020; Smith et al. 2016, 2020; Muñoz et al. 2020; Al-Zoughool et al. 2019; Arzuaga et al. 2019; Goodman et al. 2018; Guyton et al. 2018a, 2018b, 2018c; Krewski et al. 2019; Luderer et al. 2019; Nicole 2020; Temkin et al. 2020; Vandenberg et al. 2020). Those publications were evaluated for conceptual clarity, empirical transparency, susceptibility to bias, and consistency with principles of endocrine pharmacology and dose-dependence of endocrine MoAs. We found the following deficiencies in the KC approach for EDCs:

- Fails to apply the consensus definition of EDC and is not amenable to empirical testing or validation.
- Is flexible according to diverse goals, which also ensures inconsistent and unreliable results.
- Ignores principles of hormone action, characteristics of dose–response in endocrine pharmacology and toxicology, and the potential for reversibility of endpoint responses.
- Lacks a means of distinguishing endocrine-mediated from non-endocrine mediated mechanisms.
- Lacks a means to reach a negative conclusion about a chemical’s EDC properties and appears to be incapable of distinguishing EDCs from non-EDCs.
- Provides no means for developing a valid consensus among experts nor provides a means of resolving conflicting interpretations of data.

WoE methods, in contrast, search for and organize data according to specific, testable hypotheses regarding potential endocrine MoAs, appropriately contextualize in vitro and in vivo assays and evaluate data quality and relevance for each hypothesis (Rhomberg et al. 2010; Borgert et al. 2011, 2014; Rhomberg 2014; Lutter et al. 2015; Bridges and Solomon 2016; Mihaich et al. 2017; Neal et al. 2017; Mihaich and Borgert 2018; Borgert 2022). By comparison, the KC approach provides less usable information than WoE methods for the purpose of searching and organizing data. WoE methods address the need to establish causality between mechanistic steps and biological effects, which is implicit in the definition of an EDC (World Health Organization [WHO] 2002). They accomplish that by considering the mechanistic potency of chemicals relative to endogenous hormones (Borgert et al. 2018), the dose-dependence of those mechanisms and their coherence with the pattern of effects produced, and their relevance in the context of human and wildlife exposures (Borgert et al. 2011, 2014; Bridges and Solomon 2016). By comparison, the KC approach does not address that requirement and cannot determine whether chemicals meet the definition of EDC.

Contrary to the assertions of some critics, WoE evaluations can often be completed quickly and efficiently by experts in endocrine toxicology when the relevant data are available from high-quality publications or reports, obviating any perceived need to sacrifice rigor and accuracy for speed. WoE methods also reduce the likelihood of arbitrarily concluding that a chemical is an EDC, which is a significant deficiency of the KC approach. Instead of shortcuts like the KC approach, which are prone to bias, error, and arbitrary conclusions, identifying EDCs should rely on WoE evaluations that supply the critical components and scientific rigor lacking in the proposed KCs for EDC. In cases where the available data are insufficient for a robust WoE evaluation, the solution should be to generate better data, not to adopt inadequate assessment methods like the KC approach.
